# Characterization of hepatocellular adenoma and carcinoma using microRNA profiling and targeted gene sequencing

**DOI:** 10.1371/journal.pone.0200776

**Published:** 2018-07-27

**Authors:** Jian Zheng, Eran Sadot, Joana A. Vigidal, David S. Klimstra, Vinod P. Balachandran, T. Peter Kingham, Peter J. Allen, Michael I. D’Angelica, Ronald P. DeMatteo, William R. Jarnagin, Andrea Ventura

**Affiliations:** 1 Department of Surgery, Memorial Sloan Kettering Cancer Center, New York, New York, United States of America; 2 Department of Surgery, Rabin Medical Center, Petah Tikva, Israel; 3 Faculty of Medicine, Tel Aviv University, Tel Aviv, Israel; 4 Cancer Biology and Genetics Program, Memorial Sloan Kettering Cancer Center, New York, New York, United States of America; 5 Laboratory of Biochemistry and Molecular Biology, National Cancer Institute, Bethesda, Maryland, United States of America; 6 Department of Pathology, Memorial Sloan Kettering Cancer Center, New York, New York, United States of America; Taipei Medical University College of Medicine, TAIWAN

## Abstract

**Background:**

Hepatocellular adenomas (HCA) are benign liver tumors that may transform into hepatocellular carcinoma (HCC), but the molecular drivers of this transformation remain ill-defined. This study evaluates the molecular changes in HCA and HCC and in comparison to their adjacent non-neoplastic liver.

**Methods:**

11 patients with HCA and 10 patients with HCC without underlying hepatitis or cirrhosis were included in this pilot study. Tumor and non-tumor liver tissues were selected for immunohistochemical staining, small RNA sequencing, and targeted gene sequencing. We compared microRNA expressions and mutations between HCA and HCC and non-neoplastic liver.

**Results:**

HCA were classified as inflammatory (n = 6), steatotic (n = 4), or β-catenin activated (n = 1) subtypes. MicroRNA profile of all 3 HCA subtypes clustered between that of normal liver and HCC in principal component analysis. In both HCA and HCC, miR-200a, miR-429, and miR-490-3p were significantly downregulated compared to normal liver, whereas miR-452, miR-766, and miR-1180 were significantly upregulated. In addition, compared to HCA, HCC had significantly higher expression of members of the chromosome 19 miRNA cluster (C19MC), including miR-515-5p, miR-517a, miR-518b, and miR-520c-3p.

**Conclusions:**

This study indicates that while there are significant differences in the molecular profile between HCA and HCC, several miRNAs are similarly deregulated in HCA and HCC compared to adjacent normal liver. These results may provide insights into the drivers of hepatocarcinogenesis and warrant further investigations.

## Introduction

Hepatocellular adenomas (HCA) are rare, benign tumors from proliferation of hepatocytes in an otherwise normal-appearing liver, whereas hepatocellular carcinomas (HCC) are common, malignant tumors that frequently develop in a cirrhotic background [1–3]. HCA may transform into HCC, but the natural history of this progression is not well defined. In patients with underlying liver parenchymal disease, “carcinogenic field effect” has been proposed to explain the development of HCC [1, 4]. This field effect is typically associated with conditions that result in diffuse parenchymal involvement, such as hepatitis B and C infection, chronic alcohol abuse, and nonalcoholic steatohepatitis [4, 5].

In an attempt to better understand the drivers of transformation of HCA to HCC, we sought to explore unique molecular events in the adenoma-carcinoma sequence. To do so, we selected a group of HCA patients as well as patients with HCC but without hepatitis or cirrhosis, thereby excluding the “carcinogenic field-effect”-associated molecular alterations that may not be directly related to the adenoma-carcinoma sequence. However, despite the absence of background liver disease, it has been suggested that approximately 5 to 10% of pre-existing HCA may transform into HCC [6, 7]. HCA can also be difficult to be distinguished from well-differentiated HCC, and thus it is important to characterize these tumors molecularly as their prognosis and treatments are very different [8, 9].

There are 3 major molecular classifications of HCA: (a) β-catenin (CTNNB1)-activated HCA (B-HCA), (b) hepatocyte nuclear factor 1 α (HNF1α)-inactivated or steatotic HCA (H-HCA), and (c) inflammatory HCA (I-HCA) [6, 8, 9]. About 10–15% of HCA are B-HCA, 35–50% are H-HCA, 40–55% are I-HCA, while about remaining 10% of HCAs are currently unclassified [6, 8, 9]. B-HCAs are known to harbor the highest risk of malignant transformation, whereas I-HCA and H-HCA reported to have a lower risk of malignancy [6, 9, 10]. Ten percent of I-HCA may also have β-catenin-activation and all 3 subtypes of HCA may also co-occur in the same patient, albeit rarely[11]. A recent whole-exome sequencing study suggested β-catenin mutation as in B-HCA and some HCC as an early alteration, while TERT promoter mutations appear to be associated with the later steps of the progression from adenoma to carcinoma[12]. Among HCC, TERT promoter mutations are frequent in early stage HCC, whereas TP53 mutations appear at a later stage [13, 14].

In addition to studying somatic mutations in HCA and HCC through targeted gene sequencing, we also explored changes in microRNA (miRNA) expression. MicroRNAs are short (~21 nucleotide-long) noncoding RNAs that repress the expression of target genes by binding to 3’ untranslated region (3’UTR) of their transcripts and inducing their degradation and/or inhibiting their translation [15]. Because of their ability to modulate the expression of hundreds of genes, miRNAs control a multitude of biological processes and are key players in cancer pathogenesis. Although many studies have examined the expression of miRNAs in HCC, demonstrating for example increased expression of miR-21 and miR-34a and decreased expression of miR-29 and miR-122 [16–18], little is known regarding miRNA dysregulation in HCA [19].

This study compares miRNA and genetic alterations between HCA and HCC, as well as their adjacent non-neoplastic liver, in patients without underlying hepatitis or cirrhosis.

## Material and methods

### Patient and sample selection

Patient data and specimens were collected with approval of the Memorial Sloan Kettering Cancer Center (MSKCC) Institutional Review Board (protocol HBS2013103). All patients had previously provided written consent to an institutional tissue procurement protocol for research. Eleven patients with HCA and 10 patients with HCC who underwent resection between 1999 and 2013 were included in this pilot study. None of the 21 patients had viral hepatitis, prior liver-directed therapy, or any evidence of cirrhosis. None of the HCC had fibrolamellar variant. Clinical and pathological information was collected through retrospective review of the medical records.

Tumors were micro-dissected to isolate the area of interest before storage. All tissue samples were stored both as formalin-fixed paraffin-embedded (FFPE) blocks and snap-frozen in liquid nitrogen and stored at -80°C [20]. Representative histologic slides were re-reviewed by a hepatobiliary pathologist (D.S.K.) blinded to clinical and genomic data to confirm the diagnosis and to identify areas of ≥ 90% tumor cells and adjacent normal hepatocytes.

### Immunohistochemistry

Representative formalin-fixed paraffin-embedded (FFPE) blocks were obtained for each patient and immunohistochemical studies were performed on 4-μm sections according to standard protocols. HCA samples were immunostained with the following antibodies: β-catenin (CTNNB1; BD Transduction Laboratories), liver fatty acid binding protein (LFABP; Abcam # ab7807), serum amyloid A (SAA; Dako Corp., clone mc1), C reactive protein (CRP; Millipore), and glutamine synthetase (GS; BD Transduction Laboratories, clone 6). HCA with aberrant nuclear β-catenin expression were identified as β-catenin HCA (B-HCA), whereas HCA with loss of LFABP expression were considered as steatotic HCA (H-HCA), and HCA with expression of SAA and/or CRP were classified as inflammatory HCA (I-HCA) [21]. Importantly, none of the samples that stained positive for glutamine synthetase displayed a distinct “map-like” pattern without inflammatory markers, which would suggest a diagnosis of focal nodular hyperplasia, a different benign liver tumor, rather than HCA [22].

### Small RNA sequencing

Frozen tissue was homogenized in Trizol (Ambion) and total RNA extracted according to manufacturers’ instructions. High quality total RNA (1ug) was processed using the Truseq Small RNA Sample Preparation kit (Illumina). The 3’ and 5’ modified adapters were ligated specifically onto small RNAs and amplified for 14 cycles of PCR after reverse transcription. Barcodes were also introduced during the PCR steps. After size selection, the libraries were pooled and loaded onto a Hiseq (Illumina) sequencer for an SR50 run, using the TruSeq SBS Kit v3 (Illumina). An average of 11.5 million reads per sample were generated. To measure miRNA expression levels, a custom genome file of all microRNA sequences from miRBASE with the technology-specific adapter sequences was created as previously described [23]. The sequenced reads from both tumors and adjacent liver tissues were then mapped to this database using BWA (Burrows-Wheeler Alignment). The resulting SAM files were processed to evaluate full-length hits and the total number of sequences that mapped to each microRNA-adapter hybrid in the genome was determined. The miRNA count data was then transformed to log2 scale and then scaled using quantile-normalization for statistical analysis.

### IMPACT assay

Genomic DNA from tumors and adjacent normal liver tissue was extracted using phenol/chloroform and subjected to analysis by a next-generation sequencing platform. Our institutional MSK-Integrated Mutation Profiling of Actionable Cancer Targets (MSK-IMPACT) assay was designed for targeted sequencing of all exons and selected introns of 341 oncogenes, tumor suppressor genes, and other potentially actionable targets [24–26]. IMPACT analysis was performed on liver tumors and matched normal liver tissues of 9 HCA patients and 10 HCC patients whose tissues had met quality standards (http://www.cbioportal.org/study?id=hcc_msk_venturaa_2018#summary). HCA1 and HCA9 did not have IMPACT analysis. DNA from tumor and matched normal liver samples were subjected to sequence library preparation (Kapa Biosystems) and exon capture (NimbleGen). Barcoded sequence libraries were pooled at equimolar concentrations and input into a single exon capture reaction, as previously described[24, 27]. Pooled libraries containing captured DNA fragments were subsequently sequenced on the Illumina HiSeq 2500 system as 2x100bp paired-end reads. On average, 14 million reads were generated per sample, and the average coverage was greater than 600x, with more than 98.9% of the target covered at 30x. Sequence data were demultiplexed using BCL2FASTQv1.8.3 (Illumina), and vestigial adapter sequences were removed from the 3’ end of sequence reads. Reads were aligned in paired-end mode to the hg19 b37 version of the genome using BWA-MEM. Local realignment and quality score recalibration were performed using Genome Analysis Toolkit (GATK) according to GATK best practices[28]. Samples were subjected to a series of computational quality control steps to ensure genomic concordance between tumor and normal liver specimens from the same patient.

### Statistical analysis

Continuous and categorical variables were compared using Mann-Whitney U and Fisher’s exact tests, respectively. Unsupervised clustering of normalized miRNA levels was performed using classical multidimensional scale (cmdscale) function. Differential analysis of miRNA expression levels was performed using the limma package [29]. MiRNA levels with fold changes of ≥ 1 (or ≤ -1) and p-values adjusted for false discovery rate (FDR) of ≤ 0.05 were considered significant. The log2(fold change)|>1|is a widely used threshold used to restrict the analysis to miRNA that are at least two fold up or down regulated to identify meaningful and functionally relevant differences in miRNA expressions. FDR-adjusted p-values were calculated using the Benjamini and Hochberg procedure for multiple testing corrections. Heatmaps were generated using the ‘heatmap’ function, where samples and genes were clustered by the hclust function that uses Euclidian distance. Statistical analysis and data visualization were performed using R software V2.15.0 (http://www.R-project.org) and Bioconductor packages.

## Results

### Clinicopathological characteristics and immunohistochemistry

Eleven patients with HCA were included. Their median age was 40 years (range 24–56), 91% were female, and 64% had a history of oral contraceptive use (**Table 1**). The HCC cohort included 10 patients with a median age of 73 years (range 63–81) and 40% were female. All patients developed HCA and HCC without viral hepatitis or cirrhosis.

**Table 1:** Clinical and pathological characteristics of the HCA and HCC cohorts.

	HCA (n = 11)	HCC (n = 10)
**Clinical characteristics**		
Age	40 (24–56)	73 (63–81)
Female	10 (91%)	4 (40%)
Oral contraceptives use	7 (64%)	—
Body mass index, kg/m^2^	26 (22–36)	29 (22–33)
Metabolic syndrome	4 (36%)	6 (60%)
Viral hepatitis	0 (0%)	0 (0%)
Child-Pugh score	—	5 (5–5)
MELD score	—	9 (6–12)
AFP, ng/ml	2 (1.3–6.3)	15 (1.9–92881)
**Pathological characteristics**		
Multiple nodules or satellites	5 (45%)	2 (20%)
Largest tumor size, cm	5.35 (2.4–11.5)	9.2 (3.2–17)
Differentiation		
- Well	—	(10%)
- Moderate	—	(80%)
- Poor	—	1 (10%)
Microvascular invasion	—	5 (50%)
Liver fibrosis	1 (9%)	4 (40%)
Cirrhosis	0 (0%)	0 (0%)
Liver steatosis	3 (27%)	3 (30%)
Microscopic negative margin	—	10 (100%)
Extracapsular extension	—	1 (10%)
HCA subtypes		
- Inflammatory HCA	(54%)	—
- Steatotic HCA	(37%)	—
- β-catenin HCA	1 (9%)	0 (0%)

HCA, hepatocellular adenoma; HCC, hepatocellular carcinoma; MELD, Model for End-Stage Liver Disease; AFP, alpha-fetoprotein. Categorical variables are expressed as frequency (%). Continuous variables are expressed as median (range).

To further classify HCA into known subtypes, we performed immunohistochemical and mutational analysis to established molecular markers as previously described [8, 9, 21]. Based on these studies, we identified the following HCA subgroups: I-HCA (n = 6, 54%), H-HCA (n = 4, 37%), and B-HCA (n = 1, 9%) **(Table 2)**. Specifically, tumors showing expression of inflammatory proteins such as SAA and/or CRP were classified as I-HCA (**S1 Fig**) [21]. Tumors with loss of LFABP expression and HNF1α-mutations were classified as H-HCA [21]. Tumors with aberrant nuclear β-catenin expression and CTNNB1 mutations were classified as B-HCA [21]. HCC samples were stained only for β-catenin and none of them displayed abnormal nuclear β-catenin localization. Of note, 3 I-HCA and 3 HCC were resected from patients who had mild to moderate steatosis in their adjacent liver, but none had steatohepatitis or cirrhosis, which would have attributed to the “carcinogenic field defect” causing development of HCC.

**Table 2:** HCA were classified based on immunohistochemical staining with support of mutations.

ID	HCA Type	CRP	SAA	LFABP	β-catenin	GS	Mutations
**HCA1**	I-HCA	Pos	Pos	Pos	Neg	Pos	Not performed
**HCA2**	H-HCA	Neg	Pos	Neg	Neg	Neg	HNF1α
**HCA3**	I-HCA	Pos	Neg	Pos	Neg	Neg	GNAS
**HCA4**	I-HCA	Pos	Neg	Pos	Neg	Neg	FAT1, HGF
**HCA5**	I-HCA	Pos	Neg	Pos	Neg	Neg	PIK3CA, MLL2
**HCA6**	I-HCA	Pos	Pos	Pos	Neg	Pos	None
**HCA7**	H-HCA	Neg	Neg	Neg	Neg	Pos	HNF1α
**HCA8**	H-HCA	Neg	Neg	Neg	Neg	Neg	HNF1α
**HCA9**	H-HCA	Neg	Neg	Neg	Neg	Pos	Not performed
**HCA10**	I-HCA	Pos	Pos	Pos	Neg	Pos	None
**HCA11**	B-HCA	NP	NP	NP	Pos	NP	CTNNB1

HCA, hepatocellular adenoma; I-HCA, inflammatory HCA; H-HCA, steatotic HCA; B-HCA, β-catenin activated HCA; CRP, C-reactive protein; SAA, serum amyloid A; LFABP, liver fatty acid binding protein; GS, glutamine synthetase; Pos, positive; Neg, negative

### Mutational status of HCA and HCC

Analysis of IMPACT assay identified 7 different mutations among HCA tumors and 41 different mutations among HCC tumors. The assay revealed that the most frequently mutated gene in our HCA cohort was HNF1α (3/9, as 2 HCA patients did not have IMPACT analysis). HCA with HNF1α mutation, all of which belonged to the H-HCA subtype, stained negative for LFABP. A single HCA found to have a mutation in CTNNB1 and displayed abnormal β-catenin nuclear staining, was classified as B-HCA. In addition, we identified individual HCAs with mutations in GNAS, FAT1, HGF, PIK3CA, and MLL2 (**Table 2**). Within the HCC cohort, the most common mutations were found in TERT (7/10), followed by TP53 (3/10), CTNNB1 (2/10), and APC (2/10). Several additional mutations were also identified in single tumors (**S1 Table**).

### Profiling of miRNAs in HCA and HCC

**Fig 1:**
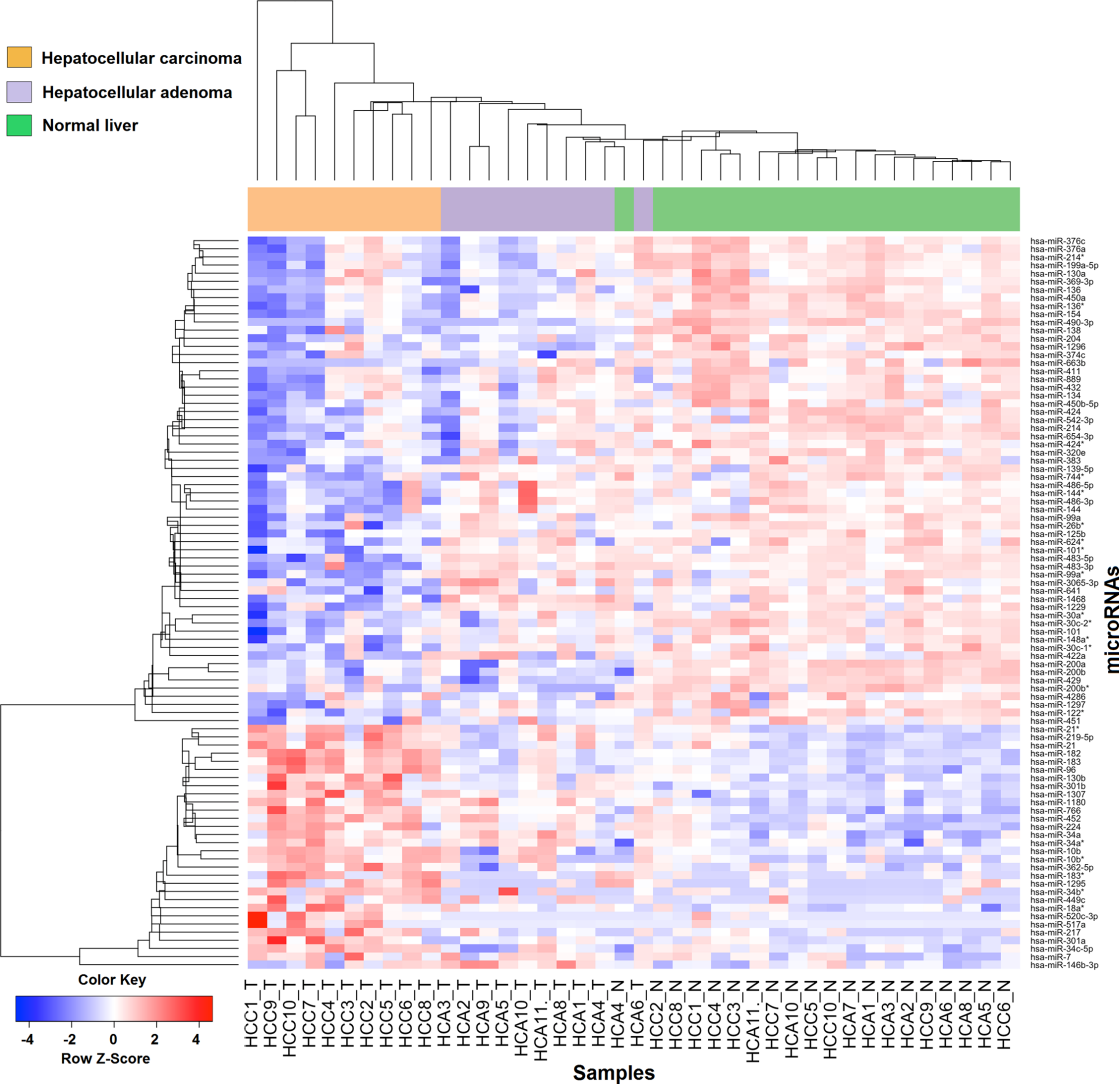
Supervised clustering showing 90 differentially expressed microRNAs between HCC and normal liver and between HCA and normal liver tumors. Data are median centered (white), with the lowest and highest intensity values in blue and red, respectively.

MiRNA profile was compared between 10 patients with HCA and 10 patients with HCC, as one of the patients with HCA (HCA7 with H-HCA) did not have adequate tumor for miRNA sequencing. Unsupervised clustering of the miRNA profiles stratified by HCA and HCC and their normal liver using classical multidimensional scaling showed HCA samples clustering between HCC and normal liver in a low-dimensional space (**S2 Fig**). Supervised clustering led to identification of 90 miRNAs that were differentially expressed between HCC and normal liver and between HCA and normal liver (**Fig 1**).

**Fig 2:**
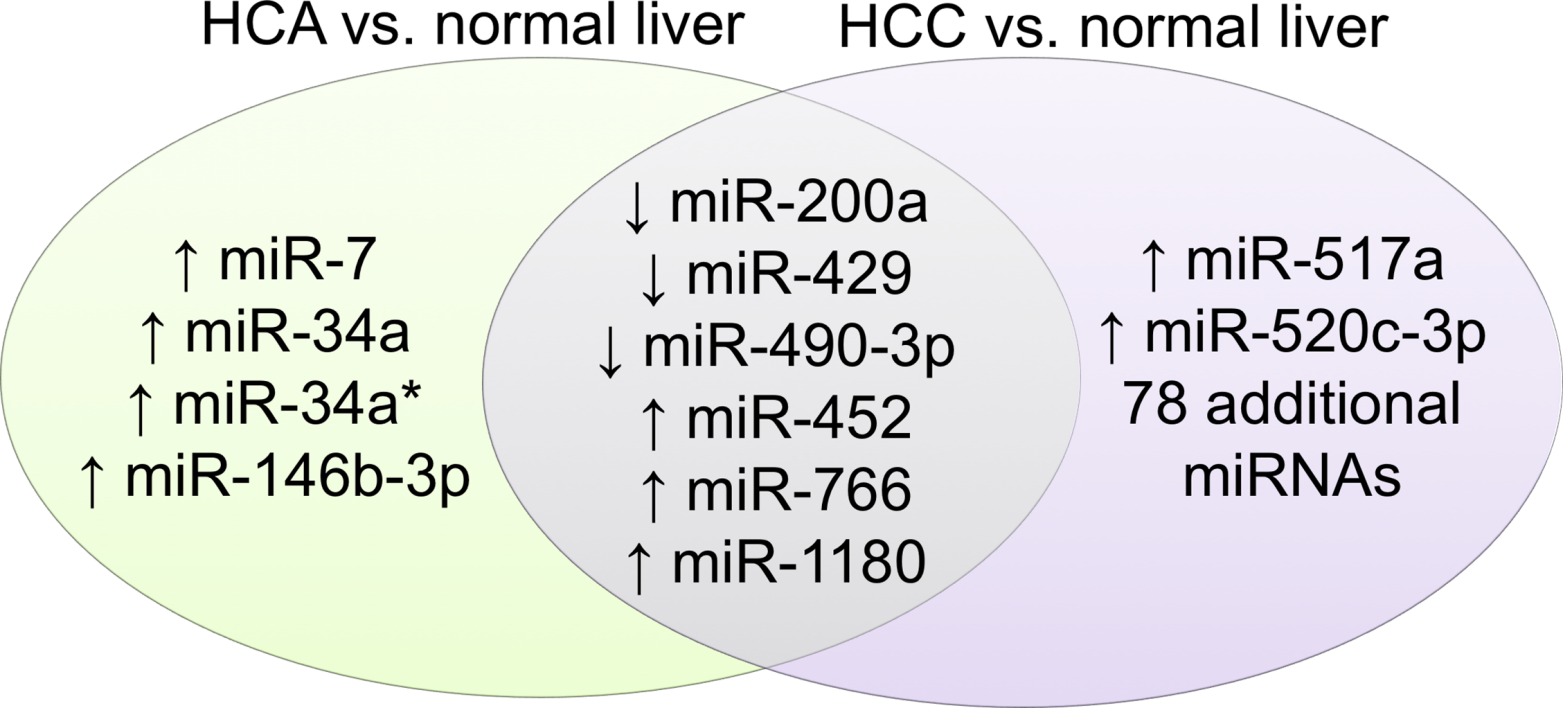
Differential expressions of miRNAs in both HCC and HCA compared to their normal liver.

A total of 86 miRNAs were significantly dysregulated in HCC compared to adjacent normal liver (unpaired analysis; adjusted p ≤ 0.05) **(S2 Table)**. Compared to HCCs, HCA samples displayed a miRNA profile that was more similar to that of adjacent normal liver, with only 10 miRNAs showing significantly different expressions (unpaired analysis; adjusted p ≤ 0.05) (**S3 Table**). Nevertheless, both HCC and HCA had significantly lower expression of miR-200a, miR-429, and miR-490-3p compared to normal liver, and had significantly higher expression of miR-452, miR-766, and miR-1180 (**Fig 2, Table 3**).

**Table 3:** Differential expressions of miRNAs in both HCC and HCA compared to their normal liver.

MiRNAs	Tumor vs normal liver	Tumor mean expression	Normal liver mean expression	Log fold change	Adjusted p-value
hsa-miR-200a	HCC (n = 10)	3.437	5.896	-2.460	0.020
	HCA (n = 10)	2.985	5.799	-2.815	0.035
hsa-miR-429	HCC (n = 10)	4.382	6.617	-2.236	0.014
	HCA (n = 10)	3.134	6.163	-3.030	0.003
hsa-miR-490-3p	HCC (n = 10)	-0.226	3.753	-3.979	0.000
	HCA (n = 10)	0.091	2.923	-2.832	0.001
hsa-miR-452	HCC (n = 10)	6.722	4.638	2.084	0.009
	HCA (n = 10)	5.748	3.496	2.251	0.018
hsa-miR-766	HCC (n = 10)	5.777	4.413	1.364	0.001
	HCA (n = 10)	5.047	3.974	1.072	0.030
hsa-miR-1180	HCC (n = 10)	5.849	4.263	1.586	0.008
	HCA (n = 10)	5.359	3.843	1.516	0.038

HCA, hepatocellular adenoma; HCC, hepatocellular carcinoma

**Fig 3:**
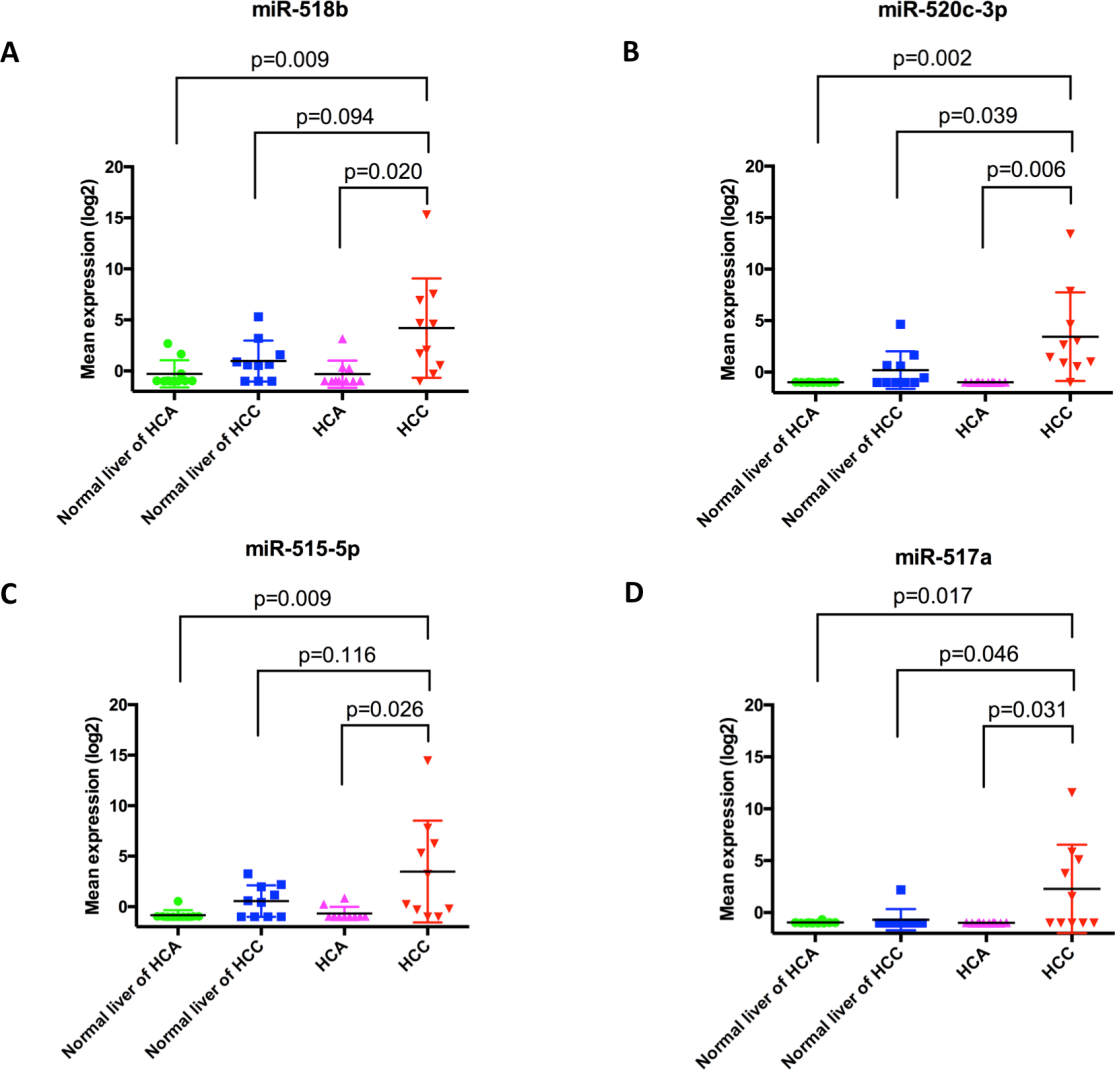
HCC had significantly higher mean expression of chromosome 19 miRNA cluster than HCA and normal liver adjacent to HCA in (A) miR-518b, (B) miR-520c-3p, (C) miR-515-5p, and (D) miR-517a. Horizontal bars represent mean value +/- standard deviation.

HCA were significantly different from HCC in the expression of 57 miRNAs (unpaired analysis; adjusted p ≤ 0.05) (**S4 Table**). Notably, selected components of the chromosome 19 miRNA cluster (C19MC), including miR-515-5p, miR-517a, miR-518b, and miR-520c-3p, were amongst the top miRNAs differentially expressed between HCC and HCA or adjacent liver samples, with levels 3 to 4 folds higher in HCC samples (**Fig 3A–3D**).

## Discussion

Although the majority of patients with HCA do not harbor malignancy, some patients will develop HCC not related to underlying liver disease [6]. Male gender, large tumor size, and B-HCA subtype have been associated with higher risk of transformation, but details of the underlying molecular events remain to be elucidated [6, 30]. None of 11 patients with HCA in this study had malignant transformation; however, the time frame for malignant change is unclear, and the question remains if transformation would have occurred with a longer follow up. The results of the present study suggest that this could be the case, given the overlap of the molecular changes between HCA and HCC that may explain progression in the hepatocellular adenoma-carcinoma sequence.

In this study, the most common mutations found among HCC were TERT (7/10), followed by TP53 (3/10), and CTNNB1 (2/10), which were consistent with prior report [31]. TERT promoter regulates telomere length and affects cellular division, whereas CTNNB1 is part of the adherens junctions that anchors actin cytoskeleton important for contact inhibition, and TP53 is a tumor suppressor that induces apoptosis [31]. CTNNB1 mutation was previously suggested as an early step in the oncogenesis and TERT promoter mutation as a later step in the transformation of HCA to HCC [12]. Our study supports these findings in that none of the patient with HCA harbored TERT mutation, whereas both HCC patients with CTNNB1 mutations also had TERT mutations. While HCA with β-catenin activated mutations (B-HCA) have shown to carry an increased risk of malignant transformation, B-HCA in this study did not reveal more similar miRNA profile to HCC than other HCA subtypes but there was only one B-HCA sample. It has been difficult to increase B-HCA sample size for this study because resection for HCA is very rare. Given the limitations of small sample size, all HCA tumors were pooled together as a group and were compared with HCC and their adjacent liver tissue instead of analysis by each of the 3 HCA subtypes. HCA tumors as a group and HCC tumors had significantly lower expression of miR-200a, miR-429, and miR-490-3p, and higher levels of miR-452, miR-766, and miR-1180, compared to their normal liver counterparts.

A role for the miR-200 family, including miR-200a and miR-429, in controlling epithelial mesenchymal transition (EMT) and metastasis is well established, including in the context of HCC [32, 33]. A prior study found decreased miR-200a level in the poorly differentiated HCC cell lines compared to well differentiated cell lines, and also showed that overexpression of this miRNA was sufficient to impair cell migration and impede EMT [32]. Another study found that miR-429 was downregulated in patients with HCC, also inhibited cell mobility by suppressing RhoA and ROCK2 mediated cytoskeletal reorganization and hindered lung metastasis in a mice model [33].

In this study, we found decreased expression of miR-490-3p in HCC and HCA compared to normal liver, which is consistent with the finding of one report but at odds with another [34, 35]. This inconsistency warrants further studies, although there is substantial evidence supporting a tumor suppressive role of miR-490-3p in other cancers, including lung, gastric, breast, and ovarian cancers [36–39].

Both HCA and HCC had significantly higher expression levels of miR-452 and miR-1180, both of which have been proposed to act as oncogenic miRNAs in HCC [40, 41]. MiR-452 and miR-1180 were shown to promote cell proliferation by targeting cyclin-dependent kinase inhibitor 1B and TNFAIP3 interacting protein 2, respectively [40, 41]. While these miRNAs were studied in HCC models, it is likely that their cancer promoting or suppressive function are analogous in HCA.

MiR-766 was also found to be upregulated in both HCA and HCC compared to normal liver. MiR-766 overexpression has not yet been implicated in HCC or HCA, but it had been implicated in other cancers. MiR-766 was found to promote cell proliferation by targeting SOX6 in colorectal cancer, and its increased expression was associated with worse overall survival in patients with lung adenocarcinoma [42, 43].

Members of a cluster of miRNAs on chromosome 19 miRNA (C19MC), including miR-515-5p, 517a, 518b, and 520c-3p, were significantly higher in HCC compared to HCA. C19MC is the largest miRNA cluster identified thus far, harboring 46 pre-miRNAs, and is located on chr19q13.41 [44, 45]. These miRNAs are expressed in undifferentiated cells and may promote oncogenesis by controlling cell differentiation [46–48]. A role for C19MC in primitive neuroectodermal brain tumors and breast cancer has also been proposed[48, 49], with reports that overexpression of miR-517a and miR-520c increases cell proliferation, migration, and invasion in HCC cell lines and promoted metastasis in a mouse model[44]. C19MC was also significantly overexpressed among hepatitis B and C related HCC compared to cirrhotic parenchyma, and overexpression of these miRNAs was associated with higher recurrence rate and shorter overall survival [45]. The mechanism for overexpression of these miRNAs in HCC is unclear, as one study suggested amplification of 19q13.41 as the primary mechanism but another study showed that aberrant hypomethylation was a more frequent event [44, 45]. Because our MSK-IMPACT panel does not cover the genes in 19q13.41, we were unable to determine copy number changes in this locus.

In this pilot study, we offer an unique perspective to evaluate and describe molecular changes in patients with HCA or HCC without underlying hepatitis or cirrhosis, thereby removing potential “carcinogenic field effect” [4]. A major limitation of this study is its small sample size, as all 3 HCA subtypes were pooled as a group when compared with HCC and their adjacent liver tissue instead of analysis by each of the 3 HCA subtypes. Resection for HCA is very rare and thus our sample size is small despite of the high-volume liver surgery in the institution over the past decades. However, in the sub-analysis of the 6 upregulated or downregulated miRNAs (miR-200a, miR-429, miR-490-3p, miR-452, miR-766, and miR-1180) for each of the 3 subtypes, their miRNA expressions were similarly up or downregulated for all 3 subtypes. This sub-analysis was not included in the manuscript as it lacks statistical power to achieve significant adjusted p-value for each HCA subtype given small sample size.

In conclusion, this study revealed while there are significant differences in the molecular profile between HCA and HCC, several miRNAs are similarly deregulated and are offering some support for progression in the hepatocellular adenoma-carcinoma sequence. In addition, HCC had higher expression of C19MC compared to HCA. Further validation using a larger sample size with more B-HCA samples and incorporating mechanistic investigations are warranted, as these miRNAs alterations have the potential to be used as surrogate marker to predict transformation from HCA to HCC.

## Supporting information

S1 Fig Representative hematoxylin and eosin stain of HCA2 (A) with negative liver fatty acid binding protein (LFABP) stain (B) revealed that it is a steatotic HCA. Representative hematoxylin and eosin stain of HCA6 (C) with concomitant positive serum amyloid A (SAA) stain (D) and positive C-reactive protein (CRP) stain (E) revealed that it is an inflammatory HCA.
(TIFF)

S2 Fig Unsupervised clustering of normalized miRNA levels was performed using classical multidimensional scale (cmdscale) function. The x and y axis represent projections of the distances between the samples.
(TIF)

S1 Table List of 7 different mutations identified among HCA and 41 different mutations identified among HCC. (PDF)

S2 Table List of 86 miRNAs that were significantly dysregulated in HCC compared to their adjacent normal liver in unpaired analysis. (PDF)

S3 Table List of 10 miRNAs that were significantly dysregulated in HCA compared to adjacent normal liver in unpaired analysis. (PDF)

S4 Table List of 57 miRNAs that were differentially expressed in HCC compared to HCA. (PDF)
