# Supplementary figures and images for: Characterization of hepatocellular adenoma and carcinoma using microRNA profiling and targeted gene sequencing
Source: PLoS One. 2018 Jul 27;13(7):e0200776. doi: 10.1371/journal.pone.0200776 (PMC6063411; doi:10.1371/journal.pone.0200776)

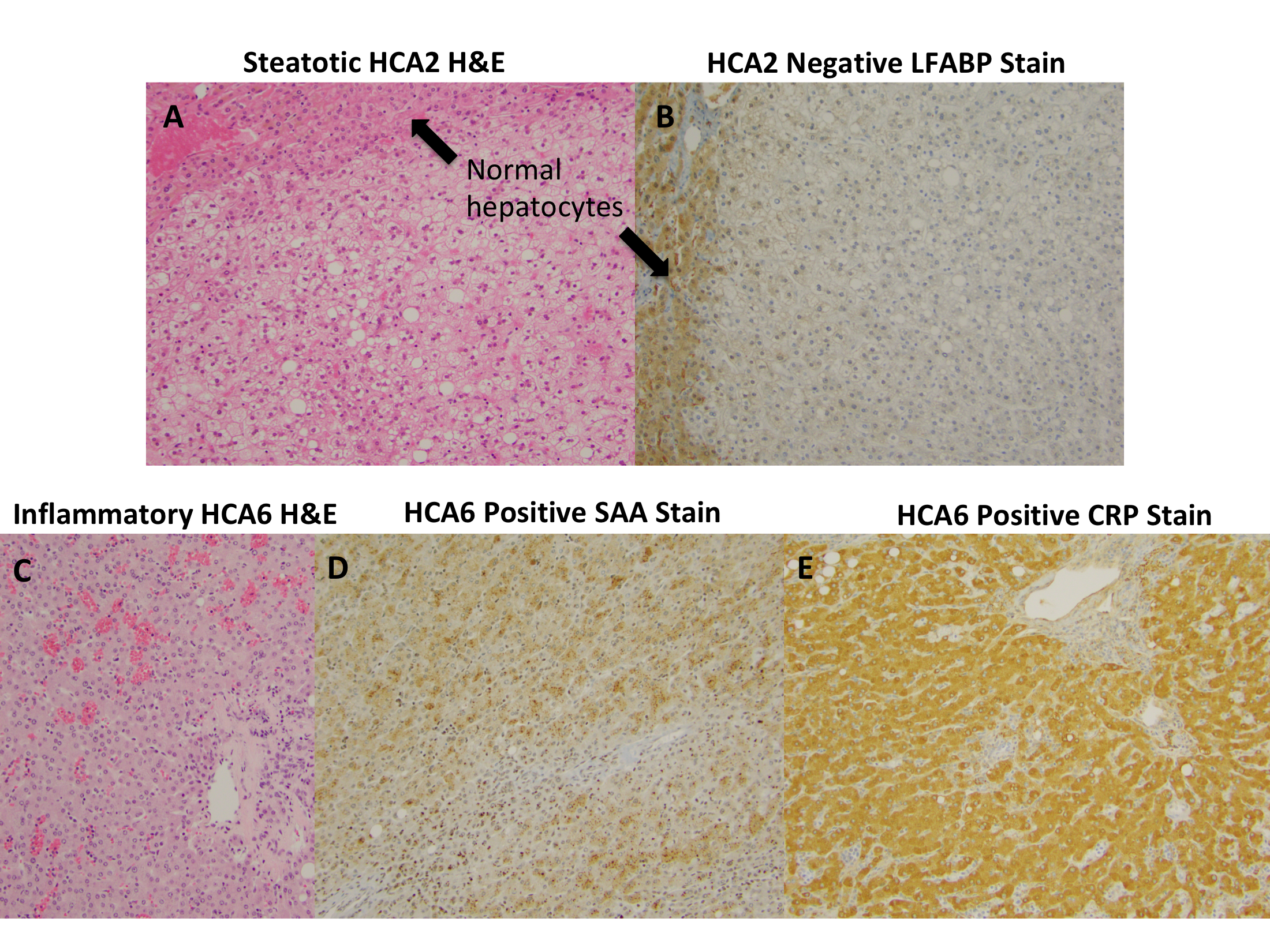

Supplement: S1 Fig — Representative hematoxylin and eosin stain of HCA2 (A) with negative liver fatty acid binding protein (LFABP) stain (B) revealed that it is a steatotic HCA. Representative hematoxylin and eosin stain of HCA6 (C) with concomitant positive serum amyloid A (SAA) stain (D) and positive C-reactive protein (CRP) stain (E) revealed that it is an inflammatory HCA. (TIFF) [file pone.0200776.s001.tiff]

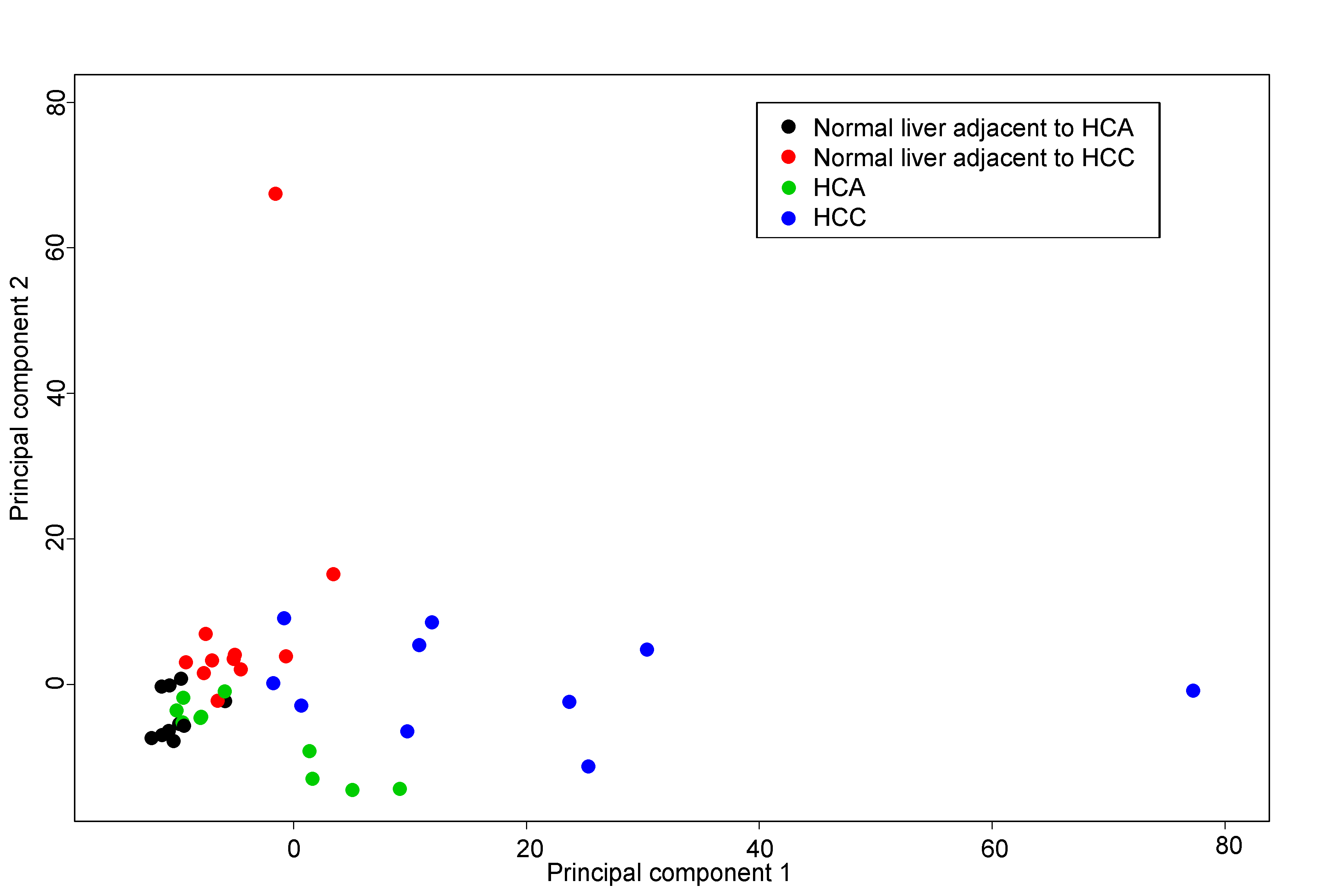

Supplement: S2 Fig — The x and y axis represent projections of the distances between the samples. (TIF) [file pone.0200776.s002.tif]
