# Supplementary material for: Characterization of hepatocellular adenoma and carcinoma using microRNA profiling and targeted gene sequencing
Source: PLoS One. 2018 Jul 27;13(7):e0200776. doi: 10.1371/journal.pone.0200776 (PMC6063411; doi:10.1371/journal.pone.0200776)
Supplement: S1 Table — (PDF) [file pone.0200776.s003.pdf]

S1 Table. List of 7 different mutations identified among HCA and 41 different mutations identified among HCC.

HCA ID	HCA Type	Mutations
**HCA1**	I-HCA	Not performed
**HCA2**	H-HCA	HNF1α
**HCA3**	I-HCA	GNAS
**HCA4**	I-HCA	FAT1, HGF
**HCA5**	I-HCA	PIK3CA, MLL2
**HCA6**	I-HCA	None
**HCA7**	H-HCA	HNF1α
**HCA8**	H-HCA	HNF1α
**HCA9**	H-HCA	Not performed
**HCA10**	I-HCA	None
**HCA11**	B-HCA	CTNNB1

HCC ID	Mutations
**HCC1**	TERT, TP53, CUL3, TET2
**HCC2**	TERT, TP53, MLL3, KEAP1, ARID2
**HCC3**	PIK3CA, PAK7, NOTCH3
**HCC4**	PHOX2B, PAX5, SUFU
**HCC5**	TERT, PIK3CG, DNMT3B, ERG
**HCC6**	TERT, CTNNB1, ARID1A, AF3B1, LATS1, CDK12, KDM6A, RBM10, CDKN1B, EP300, SF3B1
**HCC7**	TERT, CTNNB1, TP53, CTCF, BRCA1
**HCC8**	TERT, APC, ASXL2, EED, ATM
**HCC9**	SETD2, WT1, RB1, PTPRT, TMPRSS2, BCOR
**HCC10**	TERT, APC, CDKN1A, NSD1, BTK, MSH6

HCA, hepatocellular adenoma; I-HCA, inflammatory HCA; H-HCA, steatotic HCA; B-HCA, β-catenin activated HCA
