# Supplementary material for: Characterization of hepatocellular adenoma and carcinoma using microRNA profiling and targeted gene sequencing
Source: PLoS One. 2018 Jul 27;13(7):e0200776. doi: 10.1371/journal.pone.0200776 (PMC6063411; doi:10.1371/journal.pone.0200776)
Supplement: S2 Table — (PDF) [file pone.0200776.s004.pdf]

S2 Table. List of 86 miRNAs that were significantly dysregulated in HCC compared to their adjacent normal liver in unpaired analysis.

MiRNAs	HCC mean expression	Normal liver mean expression	Log fold change	Adjusted p-values
hsa-miR-182	12.293	8.517	3.776	0.000
hsa-miR-10b	13.042	9.358	3.684	0.011
hsa-miR-224	5.326	1.924	3.403	0.017
hsa-miR-183	8.234	4.931	3.302	0.000
hsa-miR-520c-3p	3.443	0.199	3.244	0.039
hsa-miR-96	6.130	3.045	3.084	0.000
hsa-miR-517a	2.279	-0.680	2.959	0.046
hsa-miR-10b*	3.492	0.672	2.820	0.011
hsa-miR-217	3.584	0.915	2.668	0.037
hsa-miR-183*	1.803	-0.622	2.425	0.006
hsa-miR-1295	1.768	-0.630	2.398	0.010
hsa-miR-301b	7.845	5.464	2.381	0.001
hsa-miR-34c-5p	5.578	3.240	2.338	0.037
**hsa-miR-452**	6.722	4.638	2.084	0.009
hsa-miR-34b*	1.531	-0.308	1.839	0.022
hsa-miR-21*	12.546	10.789	1.757	0.007
hsa-miR-219-5p	6.521	4.877	1.645	0.013
**hsa-miR-1180**	5.849	4.263	1.586	0.008
hsa-miR-18a*	4.225	2.747	1.479	0.013
hsa-miR-362-5p	7.188	5.715	1.473	0.014
hsa-miR-1307	7.325	5.915	1.410	0.002
hsa-miR-449c	0.703	-0.684	1.387	0.037
hsa-miR-130b	8.576	7.206	1.370	0.011
**hsa-miR-766**	5.777	4.413	1.364	0.001
hsa-miR-21	16.521	15.315	1.206	0.012
hsa-miR-301a	9.597	8.532	1.065	0.021
hsa-miR-432	3.907	4.908	-1.002	0.044
hsa-miR-125b	11.267	12.301	-1.035	0.009
hsa-miR-26b*	4.995	6.042	-1.047	0.025
hsa-miR-30c-1*	4.918	5.974	-1.056	0.014
hsa-miR-889	5.371	6.469	-1.099	0.046
hsa-miR-450b-5p	4.365	5.509	-1.144	0.008
hsa-miR-411	7.765	8.961	-1.196	0.048
hsa-miR-422a	13.204	14.408	-1.204	0.045
hsa-miR-424*	4.416	5.625	-1.209	0.028
hsa-miR-130a	9.006	10.224	-1.218	0.021
hsa-miR-148a*	9.906	11.132	-1.226	0.028

S2 Table, continued

MiRNAs	HCC mean expression	Normal liver mean expression	Log fold change	Adjusted p-values
hsa-miR-99a	10.007	11.254	-1.247	0.000
hsa-miR-1296	5.446	6.701	-1.256	0.022
hsa-miR-744*	2.050	3.317	-1.266	0.047
hsa-miR-1229	5.491	6.766	-1.276	0.032
hsa-miR-134	4.329	5.650	-1.321	0.037
hsa-miR-542-3p	4.604	5.943	-1.339	0.021
hsa-miR-101*	10.772	12.143	-1.371	0.017
hsa-miR-204	7.868	9.261	-1.393	0.005
hsa-miR-30a*	8.235	9.629	-1.394	0.005
hsa-miR-369-3p	4.475	5.906	-1.432	0.037
hsa-miR-320e	1.307	2.745	-1.438	0.041
hsa-miR-4286	4.908	6.350	-1.442	0.037
hsa-miR-99a*	3.484	4.929	-1.445	0.020
hsa-miR-376a	4.229	5.681	-1.452	0.016
hsa-miR-450a	5.982	7.473	-1.492	0.002
hsa-miR-641	2.088	3.614	-1.526	0.019
hsa-miR-101	11.378	12.952	-1.574	0.007
hsa-miR-486-3p	2.955	4.532	-1.578	0.021
hsa-miR-424	6.693	8.301	-1.608	0.014
hsa-miR-136*	8.292	9.913	-1.622	0.005
hsa-miR-200b*	0.619	2.243	-1.624	0.047
hsa-miR-376c	5.437	7.125	-1.688	0.011
hsa-miR-1297	3.399	5.094	-1.696	0.006
hsa-miR-624*	0.104	1.822	-1.717	0.009
hsa-miR-199a-5p	9.338	11.082	-1.744	0.025
hsa-miR-122*	7.432	9.188	-1.757	0.002
hsa-miR-144*	6.645	8.410	-1.764	0.014
hsa-miR-139-5p	4.503	6.319	-1.815	0.025
hsa-miR-214*	6.639	8.463	-1.824	0.005
hsa-miR-136	2.552	4.385	-1.833	0.009
hsa-miR-214	6.514	8.365	-1.851	0.009
hsa-miR-486-5p	11.695	13.588	-1.892	0.011
hsa-miR-30c-2*	5.407	7.316	-1.909	0.000
hsa-miR-144	7.575	9.486	-1.912	0.047
hsa-miR-1468	1.118	3.033	-1.915	0.011
hsa-miR-138	2.768	4.784	-2.015	0.007
hsa-miR-451	11.842	13.904	-2.062	0.016
hsa-miR-374c	4.888	7.007	-2.119	0.025

S2 Table, continued

**MiRNAs**	**HCC mean expression**	**Normal liver mean expression**	**Log fold change**	**Adjusted p-values**
hsa-miR-154	3.689	5.811	-2.122	0.002
**hsa-miR-429**	4.382	6.617	-2.236	0.014
hsa-miR-3065-3p	1.597	3.846	-2.248	0.020
hsa-miR-654-3p	3.266	5.522	-2.255	0.016
hsa-miR-200b	3.108	5.364	-2.256	0.044
**hsa-miR-200a**	3.437	5.896	-2.460	0.020
hsa-miR-383	0.353	2.864	-2.511	0.012
hsa-miR-663b	-0.593	1.998	-2.590	0.012
hsa-miR-483-3p	0.969	4.188	-3.219	0.001
hsa-miR-483-5p	2.824	6.106	-3.282	0.000
**hsa-miR-490-3p**	-0.226	3.753	-3.979	0.000

HCA, hepatocellular adenoma; HCC, hepatocellular carcinoma. Bolded miRNAs were also significantly dysregulated in HCA compared to normal liver.
