# Supplementary material for: Characterization of hepatocellular adenoma and carcinoma using microRNA profiling and targeted gene sequencing
Source: PLoS One. 2018 Jul 27;13(7):e0200776. doi: 10.1371/journal.pone.0200776 (PMC6063411; doi:10.1371/journal.pone.0200776)
Supplement: S3 Table — (PDF) [file pone.0200776.s005.pdf]

**S3 Table.** List of 10 miRNAs that were significantly dysregulated in HCA compared to adjacent normal liver in unpaired analysis.

| **MiRNAs** | **HCA mean expression** | **Normal liver mean expression** | **Log fold change** | **Adjusted p-values** |
|---|---|---|---|---|
| hsa-miR-34a* | 4.067 | 1.505 | 2.562 | 0.001 |
| **hsa-miR-452** | 5.748 | 3.496 | 2.251 | 0.018 |
| hsa-miR-7 | 1.559 | -0.597 | 2.156 | 0.038 |
| hsa-miR-34a | 9.670 | 7.631 | 2.039 | 0.000 |
| **hsa-miR-1180** | 5.359 | 3.843 | 1.516 | 0.038 |
| hsa-miR-146b-3p | 6.622 | 5.213 | 1.408 | 0.041 |
| **hsa-miR-766** | 5.047 | 3.974 | 1.072 | 0.030 |
| **hsa-miR-200a** | 2.985 | 5.799 | -2.815 | 0.035 |
| **hsa-miR-490-3p** | 0.091 | 2.923 | -2.832 | 0.001 |
| **hsa-miR-429** | 3.134 | 6.163 | -3.030 | 0.003 |

HCA, hepatocellular adenomas. Bolded miRNAs were also significantly dysregulated in HCC compared to normal liver.
