# Supplementary material for: Characterization of hepatocellular adenoma and carcinoma using microRNA profiling and targeted gene sequencing
Source: PLoS One. 2018 Jul 27;13(7):e0200776. doi: 10.1371/journal.pone.0200776 (PMC6063411; doi:10.1371/journal.pone.0200776)
Supplement: S4 Table — (PDF) [file pone.0200776.s006.pdf]

S4 Table. List of 57 miRNAs that were differentially expressed in HCC compared to HCA.

MiRNAs	HCC mean expression	HCA mean expression	Log fold change	Adjusted p-values
hsa-miR-518b	4.197	-0.311	4.508	0.020
hsa-miR-520c-3p	3.443	-0.984	4.427	0.006
hsa-miR-515-5p	3.472	-0.675	4.147	0.026
hsa-miR-182	12.293	8.977	3.316	0.000
hsa-miR-10b	13.042	9.727	3.315	0.027
hsa-miR-517a	2.279	-0.984	3.263	0.031
hsa-miR-96	6.130	2.971	3.158	0.000
hsa-miR-34c-5p	5.578	2.458	3.120	0.007
hsa-miR-183	8.234	5.206	3.028	0.000
hsa-miR-10b*	3.492	1.204	2.288	0.050
hsa-miR-183*	1.803	-0.333	2.136	0.015
hsa-miR-205	1.159	-0.799	1.958	0.031
hsa-miR-188-5p	1.799	-0.023	1.822	0.014
hsa-miR-301b	7.845	6.098	1.747	0.013
hsa-miR-222	6.523	5.091	1.432	0.005
hsa-miR-221	9.157	7.741	1.417	0.003
hsa-miR-362-5p	7.188	5.798	1.390	0.027
hsa-miR-19a*	5.284	3.938	1.346	0.009
hsa-miR-21*	12.546	11.219	1.326	0.050
hsa-miR-21	16.521	15.222	1.299	0.008
hsa-miR-425	8.895	7.701	1.194	0.002
hsa-miR-17	10.337	9.209	1.127	0.007
hsa-miR-93	11.284	10.175	1.110	0.002
hsa-miR-99a	10.007	11.026	-1.019	0.005
hsa-miR-29c	9.276	10.353	-1.076	0.038
hsa-miR-125b-2*	6.857	8.009	-1.152	0.002
hsa-miR-628-5p	3.631	4.869	-1.238	0.010
hsa-miR-30e	8.646	9.987	-1.341	0.020
hsa-miR-1229	5.491	6.839	-1.348	0.027
hsa-miR-101*	10.772	12.138	-1.366	0.022
hsa-miR-100*	4.035	5.462	-1.427	0.031
hsa-miR-628-3p	1.123	2.556	-1.433	0.029
hsa-miR-374a*	8.378	9.838	-1.459	0.013
hsa-miR-29b-1*	2.125	3.721	-1.596	0.027
hsa-miR-223	5.478	7.122	-1.644	0.043
hsa-miR-1827	5.267	6.978	-1.711	0.013

S4 Table, continued

MiRNAs	HCC mean expression	HCA mean expression	Log fold change	Adjusted p-values
hsa-miR-744	5.767	7.499	-1.733	0.038
hsa-miR-147b	3.953	5.694	-1.741	0.024
hsa-miR-30a	9.289	11.068	-1.779	0.022
hsa-miR-381	7.004	8.839	-1.835	0.007
hsa-miR-486-5p	11.695	13.736	-2.040	0.007
hsa-miR-144*	6.645	8.705	-2.060	0.006
hsa-miR-99a*	3.484	5.571	-2.087	0.002
hsa-miR-486-3p	2.955	5.047	-2.092	0.003
hsa-miR-383	0.353	2.528	-2.174	0.039
hsa-miR-139-5p	4.503	6.760	-2.257	0.007
hsa-miR-144	7.575	9.848	-2.274	0.022
hsa-miR-411*	5.938	8.377	-2.440	0.005
hsa-miR-1208	2.087	4.567	-2.480	0.007
hsa-miR-380	0.820	3.347	-2.528	0.004
hsa-miR-3065-5p	0.168	2.716	-2.548	0.014
hsa-miR-3065-3p	1.597	4.448	-2.850	0.004
hsa-miR-510	0.466	3.319	-2.853	0.003
hsa-miR-1468	1.118	3.975	-2.858	0.000
hsa-miR-133a	1.772	5.202	-3.431	0.020
hsa-miR-483-5p	2.824	6.313	-3.489	0.000
hsa-miR-483-3p	0.969	4.533	-3.565	0.000

HCA, hepatocellular adenomas; HCC, hepatocellular carcinoma
